# Renal Transplantation in Hepatitis C Positive Patients: A Single Centre Experience

**DOI:** 10.1155/2011/581485

**Published:** 2011-12-20

**Authors:** P. R. Shah, A. V. Vanikar, M. R. Gumber, H. V. Patel, V. B. Kute, S. M. Godara, H. L. Trivedi

**Affiliations:** ^1^Department of Nephrology and Transplantation Medicine, IKDRC-ITS, Ahmedabad 380016, India; ^2^Department of Pathology, Laboratory Medicine, Transfusion Services and Immunohematology, IKDRC-ITS, Ahmedabad 380016, India

## Abstract

*Introduction*. Hepatitis C virus (HCV) infection is an independent risk factor for renal transplantation (RTx). Immunosuppression minimization can render better quality of life to these patients. *Methods*. We analyzed 132 HCV-positive RTx patients (group A) transplanted under tolerance induction protocol (TIP) and compared them with 79 controls (group B) transplanted using standard triple drugs. TIP consisted of 1 donor-specific transfusion, peripheral blood stem cell infusion, portal infusion of bone marrow, and target-specific irradiation. Their immunosuppression was cyclosporin, 2 ± 1 mg/kg BW/day + prednisone, 10 mg/day. *Results*. TIP had no side effects. Although unequal in size, the groups were well balanced. Group A patient survival at 1, 5, and 10 years was 92.4%, 70.4%, and 63.7%, respectively, versus 75.6%, 71.7%, and 55.7% in later, and graft survival was 92.9%, 81.5%, and 79.1% versus 91.7%, 75.7%, and 67.7%, respectively. Mean serum creatinine (mg/dL) at these time periods in former was 1.38, 1.72, and 1.87, versus 1.3, 1.75, and 2.1 in later. Altered liver functions were noted in 22% patients in former versus 31% in later. Group A had lesser rejection episodes. *Conclusion*. RTx using TIP in HCV-positive patients is a viable option with acceptable outcome.

## 1. Introduction

Hepatitis C virus (HCV) infection affects 20–50% of end-stage renal disease (ESRD) patients and contributes significantly to morbidity and mortality following renal transplantation (RTx) [1, 2]. Approximately 8–28% of RTx patients die due to chronic liver disease [3]. HCV infection has a prevalence of about 2.6–66% among RTx patients in different countries with great genotype diversity in different parts of the world [3]. Antiviral drugs used for the management of HCV can have graft-threatening effects. In addition immunosuppressants themselves can be life threatening to such patients thereby putting the treating doctor in a serious dilemma, and the patient in precarious position. In such situation transplantation after adequate antiviral therapy followed by minimal immunosuppression can be a good option. 

This is a retrospective analysis of RTx carried out in our center between 1998 to 2006 in HCV-positive patients using specially designed tolerance induction protocol (TIP). Standard RTx were compared to evaluate graft function and graft/patient survival.

## 2. Materials and Methods

We analyzed medical records of HCV-positive patients (tested by third-generation enzyme linked immunoassay (ELISA)) who underwent RTx in our center from April 1998–2006 after adequate treatment. Patients were divided into 2 groups; group 1 who were transplanted after TIP with low-dose immunosuppression and group 2 who opted out of TIP and were transplanted under standard triple drug immunosuppression.

**Figure 1:**
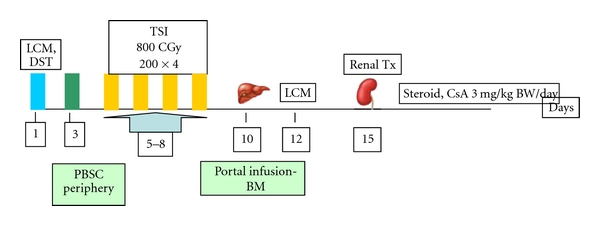
Tolerance induction paradigm for HCV-positive patients.

Written consent forms for TIP were approved by institutional review board. TIP consisted of the following steps (Figure 1).

1. HLA typing and lymphocyte cross-matching (LCM) on day 1 followed by donor-specific transfusion (DST; buffy coat infusion) in to RTx patient after bleeding 330 mL of blood from donor.
2. Mobilization of donor stem cells by leucophoresis of donor on day 3 after stimulating with granulocyte colony stimulating factor (G-CSF), 7.5 microgram/kg BW/day subcutaneously for 2 days (days 1, 2) and infusing the collected peripheral blood stem cells (PBSC) in to recipient's periphery.
3. Target-specific irradiation to subdiaphragmatic lymph nodes, spleen, part of pelvic bones, and lumbar vertebrae (200 CGY × 4 days) on days 5 to 8. Unmodified cytokine stimulated donor bone marrow (BM; 200 mL) infusion intraportally on day 10. Portal infusion technique of BM was carried out by omental vein cannulation under general anesthesia.
4. LCM on day 12.
5. RTx on day 15 with favorable LCM.
6. Peritransplant immunosuppression induction of intravenous methylprednisone, 500 mg on day before transplant, on day of transplant, 500 mg on 1st postoperative day (POD), and then switched over to oral prednisone, 30 mg/day, tapered to 10 mg/day by the end of 3 months to be continued thereafter. Cyclosporin (CsA), 3 mg/kg BW/day from day before transplant to be continued thereafter by monitoring trough levels.

No immunological preconditioning of the recipient was done. Graft-versus-host disease (GvHD) was ruled out by monitoring absence of skin rashes, gastrointestinal symptoms, abnormal liver function tests, and evidence of BM suppression.

### 2.1. HLA Typing and LCM

HLA typing and LCM were done by conventional serological technique (one lambda predot trays were used for HLA A, B, and DR typing). LCM was done by serological method using auto dithiothreitol and standard cytotoxicity methods with T and B lymphocytes each.

### 2.2. Patient Demographics

Of 211 patients studied, group A comprised of 132 patients who were transplanted under TIP with low-dose immunosuppression. Group B consisted of 79 patients, considered as controls who opted out of protocol. Demographics of both groups were fairly balanced (Table 1). Mean patient age of group A was 35.1 years with 92.4% males and in group B was 34.2 years, with 74.6% males. Mean donor age was 43.2 years in the former and 40.6 years in later. Donors were mainly parents, spouses/siblings in both groups with mean HLA match 3 ± 1.2 in former and 3 ± 1.1 in later. The commonest etiology of CRF was chronic glomerulonephritis (CGN) in both groups, with 50% patients in former and 34.2% having CGN in the later. Mean third party infusions were 13 ± 3 in former and 12 ± 3 in the later. 

### 2.3. Recipient Immunosuppression

**Group A**

CsA was the principal immunosuppressant with prednisolone, 10 mg/day. CsA doses were adjusted with an intention to maintain trough blood levels of 50–176 ngs/mL (EMIT 2000 CsA assay, USA). Mean trough levels of CsA were 180 ± 20 ng/mL in 1st 2 months of transplantation and tapered to maintain 100 ± 15 ng/mL thereafter.

**Group B**

In addition to the above drugs, group B received mycophenolate mofetil (enteric coated), 360 mg twice a day/Azathioprine, 1.5 mg/kg BW/day and doses adjusted according to BM function.

### 2.4. Rejection and Its Treatment in Both Groups

Protocol biopsies were performed at 100 days of stable graft function in subset of patients. Rejection was diagnosed on biopsy, reported as per modified Banff criteria, and treated accordingly [4, 5]. Rejections were treated with intravenous methylprednisolone, 250 mg/day × 3. Resistant rejections were treated by CsA replacement with tacrolimus in both groups. MMF/azoran was added in group A.

They were also covered with prophylaxis for CMV and pneumocystis carinii. Efficacy of protocol was tested by comparing patient and graft survival, incidence of rejections, HCV reactivation, quality of graft function, and immunosuppression requirement.

### 2.5. Statistical Analysis

Students' paired *t*-test was carried out to compare the graft function in terms of SCr, rejection episodes, and survival analysis. Survivals were examined using Kaplan-Meier analysis and compared using the log-rank test.

## 3. Results 

Side effects of G-CSF in donors were malaise, mild pyrexia, and occasional skin rashes which responded to antipyretic agents. The total average dose of CD34^+^  cells infused in group A patients was 1.3 ± 1.43 × 10^6^ cells/kg BW. Out of 132, 18 (13.6%) patients became positive on 12th day of TIP, out of them, 11 patients became negative after waiting for 8–10 days and 7 patients underwent 2 plasmapheresis and were put on MMF. They were transplanted after they became negative (after 10–15 days).

**Figure 2:**
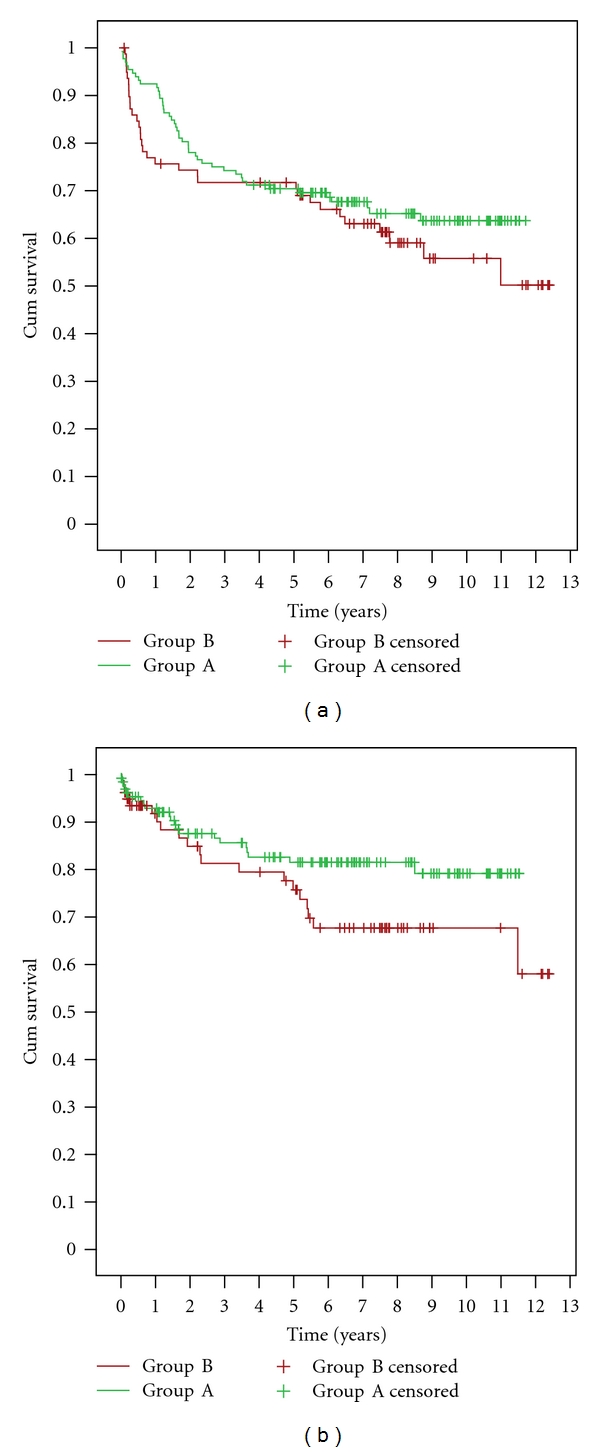
(a) Kaplan curve for patient survival functions. (b) Kaplan curve for graft survival functions.

Regarding transplantation surgery, mean donor data in both groups was similar with mean warm-ischemia time of 25 ± 10 seconds, mean anastomosis time of 30 ± 10 minutes and mean total operation time of 155 ± 20 minutes. Mean followup of group A and B was 8.38 years and 8.95 years, respectively. Mean patient survival in the former at 1, 3, 5, 7, and 10 years was 92.4%, 74.2%, 70.4%, 67.6%, and 63.7%, respectively, as compared to group B with 75.6%, 71.7%, 71.7%, 63%, and 55.7% survival, respectively. Mean graft survival in the former at 1, 3, 5, 7, and 10 years was 92.9%, 85.6%, 81.5%, 81.5%, 79.1%, respectively, as compared to group B with 91.7%, 81.2%, 75.7%, 67.7%, 67.7%, respectively. Kaplan Meier graphs of patient and graft survival are shown in Figures 2(a) and 2(b).

Graft function in terms of SCr (in mg/dL) in both groups at 1, 3, 5, 7, and 10 years was 1.38 ± 0.29, 1.55 ± 0.34, 1.72 ± 0.47, 1.8 ± 0.39, and 1.87 ± 0.69, versus 1.3 ± 0.37, 1.58 ± 0.64, 1.75 ± 0.61, 1.97 ± 0.73, and 2.1 ± 0.81 in group B. Liver function status was deranged and accompanied by presence of HCV-RNA (tested by PCR and 1 to 5 million copies/mL with mean rate of 1.65 ± 0.75 copies/mL in TIP and 2.3 ± 2.05 copies/mL in controls were noted) in 22% (*n* = 29) patients of group A out of which 8.3% (*n* = 11) succumbed to chronic liver failure and in 31% (25 patients) of group B, 16.5% (13 patients) died of chronic liver failure. Group A patients had better graft and patient survival along with graft function status as compared to group B (statistically not significant).

In terms of rejection there was statistically significant decrease in T-cell-mediated rejections and chronic changes in the former as compared to the later (Table 2). Majority of the patients responded to antirejection therapy. However 4/132 (about 3%) in TIP group and 4/79 (about 5%) in control group did not respond and eventually lost their grafts to chronic dysfunction and eventually succumbed to secondary infections and septicemia. Incidence of reactivation of HCV was also significantly less in the former as compared to controls. In TIP group totally 45 (34%) patients and in controls 32 (40.5%) were lost over a followup of 12 years. Out of these 11 in TIP and 13 in controls succumbed to liver failure, others to chronic graft dysfunction-related morbidity or to septicemia. The other advantage in group A was significantly less requirement of maintenance immunosuppression in the form of CsA, 2 ± 1 mg/kg BW and prednisone, 5–10 mg/day versus group B with standard triple drug immunosuppression.

## 4. Discussion

The effect of pretransplant HCV infection on survival of patients and grafts in RTx is controversial [6]. However, survival is better in HCV RTx patients as compared to dialysis [7].The goals of pretransplantation HCV therapy are to decrease the risk for progression of HCV-associated liver disease, stabilize renal function in patients with HCV-related glomerulopathy, and prevent development of HCV-associated disease after transplantation [8]. The use of immunosuppression predisposes RTx patients to risks of deranged liver functions and mortality [9, 10]. A meta-analysis of natural history of HCV in 6365 RTx patients showed that anti-HCV antibody was an independent risk factor for death and graft failure with relative risk of 1.79 [11]. In our center we offer TIP to all patients, and we start the protocol only after informed consent form is signed by patient, donor, and witness. However, all donors are not willing to undergo stimulation protocols, abdominal fat resection, BM aspiration, and above all they are not willing to wait till renal transplantation. We explain to them that it may take a month or little longer for transplantation, to finish the protocol and if patient becomes lymphocyte cross-match positive, waiting period can become longer. Many patients and donors cannot get leave from their work for such a long period even if they do not have to stay in hospital, they need to visit us frequently which they are not willing. With minimization of immunosuppression, patients are at lower risk of infections and hence return to better quality of life. Secondly lowering of rejection incidence and severity automatically saves financial burden, especially in India where there is no financial support from government medicare/medical insurance. With TIP, use of less number and low-dose of drugs brings down the cost, though we have not touched upon this aspect here. We have more than 10 years of experience of using TIP in about 1500 patients and hence we modified it for HCV-positive patients and implemented it [12]. Our study shows that with use of tolerance induction protocol for HCV-positive patients, quality of life, graft function, and survival are reasonably good even for a long period of ten years. Our control (group B) patients have reasonable quality of graft function and survival as found in other studies [6–11]. Interestingly, tolerance induction protocol yielded significantly less chances of reactivation of HCV as compared to controls. This could be attributed to better immune competence in these patients since they require less immunosuppression.

## 5. Conclusion

RTx is an acceptable option for HCV-positive patients with ESRD, and tolerance induction protocol is preferable over standard triple drug immunosuppression in these group of patients.

## Figures and Tables

**Table 1:** Demographics of renal transplant HCV-positive patients (group A) and control patients (group B).

Patients (*n* = 211)	Group A (*n* = 132)	Group B (*n* = 79)
Mean age (years; ± SD)	35.1 ± 11.2	34.2 ± 11.4
Mean donor age (years; ± SD)	43.2 ± 11.1	40.6 ± 11.4
Patient gender (male : female)	122 : 10	59 : 20
Third-party infusions	13 ± 3	12 ± 3
HLA match: *n*/6—in percentage		
0	12.1 (*n* = 16)	5.1 (*n* = 4)
1	18.9 (*n* = 25)	7.6 (*n* = 6)
2	25.8 (*n* = 34)	10.1 (*n* = 8)
3	30.3 (*n* = 40)	34.1 (*n* = 27)
4	5.3 (*n* = 7)	8.9 (*n* = 7)
5	1.5 (*n* = 2)	3.8 (*n* = 3)
6	0	0
Not performed	6.1 (*n* = 8)	30.4 (*n* = 24)
Basic disease—in percentage		
CGN	50 (*n* = 66)	45.6 (*n* = 36)
DM-DN	10.6 (*n* = 14)	3.8 (*n* = 3)
CTIN	14.4 (*n* = 19)	10.1 (*n* = 8)
Obstructive uropathy	4.6 (*n* = 6)	6.4 (*n* = 5)
ADPKD	1.5 (*n* = 2)	3.8 (*n* = 3)
Nephrosclerosis	12.1 (*n* = 16)	16.5 (*n* = 13)
FSGS	0	2.5 (*n* = 2)
Membranous nephropathy	0.8 (*n* = 1)	2.5 (*n* = 2)
IgA nephropathy	0	1.26 (*n* = 1)
MPGN	0	1.26 (*n* = 1)
Vasculitis	0.8 (*n* = 1)	0
HUS	0	1.26 (*n* = 1)
Lupus nephritis	2.2 (*n* = 3)	2.5 (*n* = 2)
Alport syndrome	1.5 (*n* = 2)	1.26 (*n* = 1)
Others	1.5 (*n* = 2)	1.26 (*n* = 1)

Abbreviations: ADPKD: autosomal dominant polycystic kidney disease; CGN: chronic glomerulonephritis; CTIN: chronic tubulointerstitial nephritis; DM-DN: diabetes mellitus, diabetic nephropathy; FSGS: focal segmental glomerulosclerosis; HUS: hemolytic uremic syndrome; MMF: mycofenolate mofetil; MN: membranous nephropathy.

**Table 2:** Results.

Patients (*n* = 211)	Group A (*n* = 132)	Group B (*n* = 79)	*P* value
Study period	Jan 99–Dec 06	Jan 98–Dec 06	
Mean followup (years; range)	8.38 ± 2.2 (3.8–11.8)	8.95 ± 2.2 (4–12.6)	
Mean SCr (mg/dL) at			
1 year	1.38 ± 0.29	1.3 ± 0.37	NS
3 years	1.55 ± 0.34	1.58 ± 0.64	NS
5 years	1.72 ± 0.47	1.75 ± 0.61	NS
7 years	1.8 ± 0.39	1.97 ± 0.73	NS
10 years	1.87 ± 0.69	2.1 ± 0.81	NS
Predominant biopsy findings—percentage			
Acute rejection episodes—percentage			
Bcell mediated	8.3 (*n* = 11)	15.2 (*n* = 12)	NS
Tcell mediated	8.3 (*n* = 11)	17.7 (*n* = 14)	0.03
Suspicious T/B	9.8/0 (*n* = 13/0)	20.2/5 (*n* = 16/4)	0.024/0.022
Acute CNI toxicity	12.1 (*n* = 16)	19 (*n* = 15)	NS
Recurrence	1.5 (*n* = 2)	2.5 (*n* = 2)	NS
Chronic rejections			
Bcell mediated	3 (*n* = 4)	6.3 (*n* = 5)	NS
Tcell mediated	3.8 (*n* = 5)	11.4 (*n* = 9)	0.029
IFTA	8.3 (*n* = 11)	19 (*n* = 15)	0.018
Chronic CNI toxicity	9.1 (*n* = 12)	16.5 (*n* = 13)	NS
Recurrence	2.3 (*n* = 3) (ATIN)	3.8 (*n* = 2 (ATIN), *n* = 1 (MPGN))	NS
De novo nephropathy	1 (MN)	0	NS
Chronic liver failure dueto reactivation	22 (*n* = 29)	31 (*n* = 25)	*P* = 0.0002

Abbreviations: CNI: calcineurin inhibitor; CGN: chronic glomerulonephritis; CsA: cyclosporin A; ELISA: enzyme linked immunoassay; ESRD: end-stage renal disease; G-CSF: granulocyte colony stimulating factor; HCV: hepatitis C virus; IFTA: unexplained interstitial fibrosis and tubular atrophy; LCM: lymphocyte cross-matching; PBSC: peripheral blood stem cells; POD: postoperative day; RTx: renal transplantation; SCr: serum creatinine; TIP: tolerance induction protocol.
